# Longitudinal changes in brain metabolites following pediatric concussion

**DOI:** 10.1038/s41598-024-52744-7

**Published:** 2024-02-08

**Authors:** Parker L. La, Robyn Walker, Tiffany K. Bell, William Craig, Quynh Doan, Miriam H. Beauchamp, Roger Zemek, Keith Owen Yeates, Ashley D. Harris, Pediatric Emergency Research Canada A-CAP study team, Keith Owen Yeates, Miriam H. Beauchamp, Bruce H. Bjornson, Jocelyn Gravel, Angelo Mikrogianakis, Bradley Goodyear, Nishard Abdeen, Christian Beaulieu, Mathieu Dehaes, Sylvain Deschenes, Ashley D. Harris, Catherine Lebel, Ryan Lamont, Tyler Williamson, Karen Maria Barlow, Francois Bernier, Brian L. Brooks, Carolyn Emery, Stephen B. Freedman, Kristina Kowalski, Kelly Mrklas, Lianne Tomfohr-Madsen, Kathryn J. Schneider

**Affiliations:** 1 Department of Radiology, University of Calgary, Calgary, AB Canada; 2 Alberta Children’s Hospital Research Institute, Calgary, AB Canada; 3 Hotchkiss Brain Institute, University of Calgary, Calgary, AB Canada; 4 Department of Pediatrics, Stollery Children’s Hospital, University of Alberta, Edmonton, AB Canada; 5 Department of Pediatrics, BC Children’s Hospital Research Institute, University of British Columbia, Vancouver, BC Canada; 6 Department of Psychology, Ste Justine Hospital Research Centre, University of Montreal, Montreal, QC Canada; 7 Department of Pediatrics and Emergency Medicine, Children’s Hospital of Eastern Ontario, University of Ottawa, Ottawa, ON Canada; 8 Childrens’ Hospital of Eastern Ontario Research Institute, University of Ottawa, Ottawa, ON Canada; 9 Department of Psychology, University of Calgary, Calgary, AB Canada; 10 Department of Pediatrics, Ste Justine Hospital, Universite de Montreal, Montreal, Québec Canada; 11 Department of Radiology, Children’s Hospital of Eastern Ontario, University of Ottawa, Ottawa, ON Canada; 12 Department of Biomedical Engineering, University of Alberta, Edmonton, AB Canada; 13 Department of Radiology, Radiooncology and Nuclear Medicine, Ste Justine Hospital, Université de Montréal, Montreal, Québec Canada; 14 Department of Medical Genetics, Alberta Children’s Hospital, University of Calgary, Calgary, AB Canada; 15 Department of Community Health Sciences, University of Calgary, Calgary, AB Canada; 16 Department of Clinical Neurosciences, University of Calgary, Calgary, AB Canada; 17 Department of Pediatrics, Alberta Children’s Hospital, University of Calgary, Calgary, AB Canada; 18 Faculty of Kinesiology, University of Calgary, Calgary, AB Canada; 19 Research Innovation and Analytics, Alberta Health Services, Calgary, AB Canada

**Keywords:** Paediatric research, Brain injuries, Neurochemistry

## Abstract

Concussion is commonly characterized by a cascade of neurometabolic changes following injury. Magnetic Resonance Spectroscopy (MRS) can be used to quantify neurometabolites non-invasively. Longitudinal changes in neurometabolites have rarely been studied in pediatric concussion, and fewer studies consider symptoms. This study examines longitudinal changes of neurometabolites in pediatric concussion and associations between neurometabolites and symptom burden. Participants who presented with concussion or orthopedic injury (OI, comparison group) were recruited. The first timepoint for MRS data collection was at a mean of 12 days post-injury (n = 545). Participants were then randomized to 3 (n = 243) or 6 (n = 215) months for MRS follow-up. Parents completed symptom questionnaires to quantify somatic and cognitive symptoms at multiple timepoints following injury. There were no significant changes in neurometabolites over time in the concussion group and neurometabolite trajectories did not differ between asymptomatic concussion, symptomatic concussion, and OI groups. Cross-sectionally, Choline was significantly lower in those with persistent somatic symptoms compared to OI controls at 3 months post-injury. Lower Choline was also significantly associated with higher somatic symptoms. Although overall neurometabolites do not change over time, choline differences that appear at 3 months and is related to somatic symptoms.

## Introduction

Millions of children experience concussion annually^1,2^. Most children will experience symptoms acutely following injury and recover within 4 weeks^3^; however, 15–30% will go on to develop persisting symptoms that persist for longer than 3 months following injury^4^. Concussion is a heterogeneous injury and recovery trajectories vary across children, making it difficult to identify factors that predict symptom persistence.

Concussion is characterized by a metabolic cascade where ions and metabolites are in a dynamic flux^5^, suggesting neurochemistry has the potential to be disturbed following concussion^5–9^. Magnetic resonance spectroscopy (MRS) can be used to study such alterations non-invasively. The most commonly studied neurometabolites are tNAA (*N*-Acetyl Aspartate + *N*-Acetyl Aspartyl Glutamate), tCr (Creatine + Phosphocreatine), tCho (Choline + Phosphocholine + Glycero-phosphocholine), Glx (Glutamate + Glutamine), and mI (Inositol + Glycine). These metabolites are typically interpreted as markers of neuronal health, bioenergetics, membrane integrity, excitation, and inflammation, respectively^10^.

In the largest study using MRS to study pediatric concussion to date (361 concussion participants), there were no neurometabolite differences in the left dorsal lateral pre-frontal cortex (L-DLPFC) between concussion and orthopedic injury (OI) groups 12 days after injury^11^. This lack of group difference was maintained even when considering multiple covariates (e.g., participant age, time since injury, voxel-tissue composition). Similarly, a study using diffusion tensor imaging to assess white matter microstructure in pediatric concussion also failed to find differences in white matter microstructure at 11 days following injury^12^, although another study found higher mean diffusivity in the thalamic radiation 3 months post-injury and reductions in mean diffusivity of the arcuate fasciculus at 6 months post-injury^13^. Thus, examining MRS in the longer term following concussion may reveal neurometabolic changes. The current longitudinal literature is sparse and mixed, with some but not all studies reporting neurometabolite differences^14–18^. Furthermore, studies that have shown neurometabolite differences have not shown consistent results, likely because of differences in time of assessment post-injury, regions studied, heterogeneity of concussion presentation, or comparison groups. Additionally, many of the current concussion studies in pediatrics^14–18^ use a healthy control group perhaps biasing metabolite effects towards representing general injury alterations for example due to pain, rather than concussion specifically. Therefore, the use of an orthopedic injury control group is a desirable approach to detect concussion specific effects. Finally, little is known about the association between neurometabolites and persisting symptom status in pediatric concussion.

The primary aim of the current study was to determine how brain metabolites change over 6 months following concussion and whether symptom status at the 4 week timepoint impacts this trajectory. The second aim was to determine how metabolites differ between concussion and OI controls at 3- and 6 months and how those with and without symptoms at 3 and 6 months may be different in metabolites. The third aim was to determine whether metabolites are associated with symptom burden at 12 days, 3 months, and 6 months post-concussion.

## Materials and methods

### Study design and procedure

Data were collected as part of a large multisite study of pediatric concussion, Advancing Concussion Assessment in Pediatrics (A-CAP)); for a detailed description, see Yeates et al. 2017^19^. Participants were recruited from five pediatric Emergency Departments (ED) within the Pediatric Emergency Research Canada (PERC) network^20^ in Canada. Participating hospitals were Alberta Children’s Hospital (Calgary), Stollery Children’s Hospital (Edmonton), British Columbia Children’s Hospital (Vancouver), Children’s Hospital of Eastern Ontario (Ottawa), and Centre Hospitalier Universitaire (CHU) Sainte-Justine (Montreal). Participants were recruited if they presented with concussion or orthopedic injury (OI, the comparison group) within 48 h of injury. This study was approved by the research ethics board at each site (Conjoint Health Research Ethics Board at the University of Calgary, REB152296; Ste Justine Research Institute, University of Montreal, MP-21-2017-1332; CHEO REB16/23E; University of Alberta HREB, 64,780; University of British Columbia Children’s & Women’s Research Ethics Board, H16-00,104) where all research was performed in accordance with the relevant guidelines and regulations. Informed consent and assent were obtained from the parents/guardians and the youth participants respectively.

### Participants

All participants were between the ages of 8.00–16.99 years at the time of recruitment. Concussion participants sustained a blunt head trauma and presented with an observed loss of consciousness, a Glasgow Coma Scale (GCS) score of 13 or 14, and/or at least one acute sign or symptom of concussion (i.e., post-traumatic amnesia, focal neurological deficits, skull fracture, posttraumatic seizure, vomiting, headache, dizziness, or other mental status abnormalities). Children who showed delayed neurological deterioration (GCS < 13) or required neurosurgical intervention were excluded. Participants were also excluded if they had a loss of consciousness of greater than 30 min, post-traumatic amnesia of greater than 24 h, or any associated injuries with scores of greater than 4 on the Abbreviated Injury Scale^21^.

OI participants sustained an upper or lower extremity fracture, sprain, or strain due to blunt force/physical trauma resulting in an Abbreviated Injury Scale^21^ score of 4 or less. Youth were excluded from the OI group if they had any head trauma or suspicion of concussion and concussion-related symptoms at the time of recruitment, or an injury requiring surgical intervention or procedural sedation. Youth were excluded from both groups if they sustained a concussion within 3 months of recruitment. A thorough description of the inclusion and exclusion criteria and the multi-site study are described in the study protocol^17^.

### MRI and MRS

Participants completed 3 T magnetic resonance imaging (MRI) including MRS. The target for the first visit post-recruitment was about 1–2 weeks post-injury (will now be referred to as 12 days) and then participants were randomized to either a 3 or 6 month follow-up MRI. The 5 sites included 3 GE scanners (General Electric MR750w in Calgary; General Electric MR750 in Montreal and Vancouver) and 3 Siemens scanners (Siemens Prisma in Edmonton and Montreal; Siemens Skyra in Ottawa). Data acquired at 12 days was analyzed for cross-sectional changes shortly after injury with examination of possible covariate effects and previously published as La et al.^11^.

T1-weighted images were acquired. The sites that used GE scanners used a 3D T1-weighted fast spoiled gradient echo brain volume (FSPGR BRAVO) sequence with a TR/TE/TI = 8.25/3.16/600 ms with a field of view of 24 cm^2^. The sites that used Siemens scanners used a 3D T1-weighted magnetization prepared rapid acquisition gradient echo (MPRAGE) sequence with TR/TE/TI = 1880/2.9/948 ms and a field of view of 25.6 cm^2^. Acquisitions from both vendors used 192 slices, a flip angle of 10° with a voxel size of 0.8 × 0.8 × 0.8 mm^3^.

Single voxel MRS data was acquired in the left dorsolateral prefrontal cortex (L-DLPFC) using Point RESolved Spectroscopy (PRESS) localisation. The DLFPC was chosen as it is involved with many concussion symptoms owing to its involvement with executive function, and its involvement in previous MRS in concussion studies. The frontal region has also been recommended for single voxel MRS by the ENIGMA MRS Working Group for the study of traumatic brain injury^22^.

**Figure 1:**
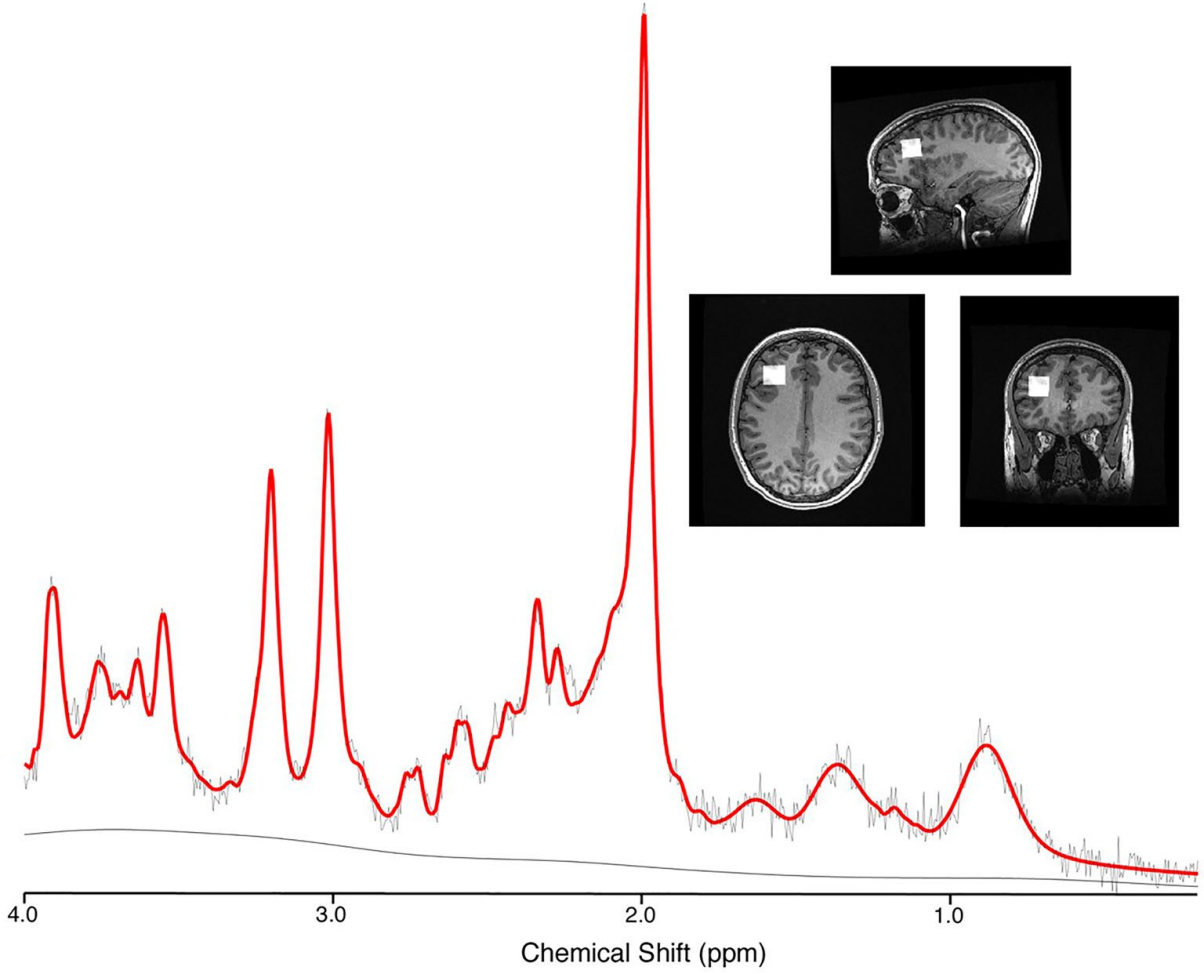
Example spectrum (black) with LCModel fit (red), and voxel placement in the left dorsal lateral prefrontal cortex (L-DLPFC).

GE sites used a 32-channel head coil while Siemens sites used a 64-channel head coil. Acquisition parameters were; TE/TR = 30/2000 ms, 96 water suppressed averages (with 8 step phase cycle for GE and 16 step phase cycle for Siemens), 8 unsuppressed water averages, 2 × 2 × 2 cm^3^ voxel, spectral width 5000 Hz (GE) or 2000 Hz (Siemens), number of points 4096 (GE) or 2048 (Siemens), water suppression methods were CHESS (GE) and WET (Siemens). Each participating site was provided with several reference images to ensure standardized voxel placement, and an example of voxel placement is shown in Fig. 1.

### Data analysis

As GE data had individual averages available, a complete pre-processing pipeline was used based on consensus recommendations^23^ and included: combination of receiver channels, removal of bad averages, retrospective shot-by-shot frequency and phase correction, left shifting, and zero order phase correction. These pre-processing steps were automated and completed using FID-A^24^. The Siemens site data were fully averaged upon scanner export, so the only pre-processing performed was by the vendor software.

Data from both GE and Siemens were quantified with LCModel version 6.3-1J^25^. Basis sets used in LCModel analyses were customized for each vendor and generated in FID-A using vendor-specific pulse shapes, spectral width, and number of points. The generated basis sets included the following metabolites: Alanine, Aspartate, *β*-Hydroxybutarate, Choline, Citrate, Creatine, Ethanol, Gamma-aminobutyric acid, Glucose, Glutamine, Glutamate, Glycine, Glycerophosphocholine, Glutathione, *myo*-Inositol, Lactate, *N*-acetyl-aspartate, *N*-acetyl-aspartyl-glutamate, Phosphocholine, Phosphocreatine, Phosphoenolamine, *Scyllo*-inositol, Taurine. The default LCModel macromolecular and lipid basis sets were also included in the quantification. The default LCModel control file was used in fitting, and subsequently the water attenuation and white matter voxel tissue content assumptions included in the LCModel default control file were corrected for in our tissue correction steps. Quality assurance steps that were undertaken included visual inspection of each spectrum, an SNR minimum threshold of at least 45 and a CRLB for each metabolite of less than 20%.

Tissue correction was performed following analysis according to recent recommendations and guidelines^23^ for metabolites in reference to water. The co-registration and segmentation functions from Gannet (Version 3.1)^26^ were used to register the MRS voxel to the corresponding anatomical image and segment it into white matter (WM), gray matter (GM), and cerebrospinal fluid (CSF). Tissue correction using literature values for T1 and T2 relaxation times and proton density was completed using the equations specified in the expert consensus in Near et al.^23^.

### Symptom measure

Parents completed the Health and Behavior Inventory (HBI)^27^, which consists of 20 Likert style questions to assess concussion symptoms and severity and provides summary scores for somatic and cognitive symptoms. Somatic symptoms include fatigue, dizziness, headaches, nausea, visual disturbance, etc. Cognitive symptoms include inattention, poor concentration, and memory deficits. For more detail on this classification and the HBI inventory please refer to Ayr et al.^27^.

The HBI has been adopted as a core measure in the common data elements for pediatric traumatic brain injury (TBI)^28^. Parents rated post-injury symptoms at the first (~ 12 days post injury), 4 week (no imaging), 3 month and the 6 month visits. Pre-injury symptoms were also rated retrospectively by parents at the first assessment. Using the pre-injury symptoms, the reliable change^29,30^ of the HBI was determined to classify the children with concussion into symptomatic and asymptomatic groups for somatic and cognitive symptoms at 4 weeks, 3 months, and 6 months. This has been validated and done in previous studies^13,30^. In the current study, symptom scores at the 4 week timepoint were used for the longitudinal trajectory analyses as this is the time to define persistent post-concussive symptoms. Additionally, cross-sectional analyses used the 3 months and 6 months symptom scores with the MRS data collected at these time points.

### Statistics

All statistical analyses were completed using IBM SPSS 26 (IBM Corp. Released 2019. IBM SPSS Statistics for MacOS, Version 26.0. IBM Corp., Armonk, NY, USA).

The first analysis used linear mixed effects models to examine whether metabolites (tNAA, tCr, tCho, Glx, mI) changed over time in concussion participants. A repeated measures linear mixed model was used to account for data loss between timepoints. A second set of linear mixed effects models then examined whether metabolites were affected by different injury groups (Asymptomatic, Symptomatic, OI) over time; injury group was determined by the reliable change at 4 weeks. A timepoint by group interaction term was included to test for changes in the different groups over time. These models included fixed effects for age and sex, while controlling for site and vendor as random effects.

The second analysis used univariate ANCOVAs to examine cross-sectional group differences in metabolites at the 3- and 6 month timepoints. Concussion participants were classified as asymptomatic or symptomatic according to the reliable change index at each timepoint studied and compared to OI. Therefore, 2 sets of analyses were done for each metabolite for 3- and 6 month metabolite data (3 month reliable change for somatic and cognitive symptoms, and 6 month reliable change for both somatic and cognitive symptoms). These models also controlled for age, sex, site, and vendor as covariates. Bonferonni corrections were applied to each individual model for the 3 groups included (somatic symptoms, cognitive symptoms, and OI.

The third analysis contained 10 sets of linear regressions for each metabolite. They all examined the association between metabolite levels and the concussion symptom ratings (2-measures of symptoms, cognitive and somatic) at 12 days, 3 months, and 6 months in concussion participants. The ten models determined the relationship between each of the following; metabolites at 12 days and symptoms at 12 days, metabolites at 12 days and symptoms at 3 months, metabolites at 12 days and symptoms at 6 months, metabolites at 3 months and symptoms at 3 months, and lastly metabolites at 6 months and symptoms at 6 months. Each model controlled for age, sex, site, and vendor as covariates. These analyses were corrected for multiple comparisons using a Bonferonni correction within the model to control for multiple metabolites present in each model.

### Ethics approval and consent to participate

The study was approved by the research ethics board at each participating site.

### Patient consent statement

Informed consent and assent were obtained from the parents/guardians and the youth participants, respectively.

## Results

**Figure 2:**
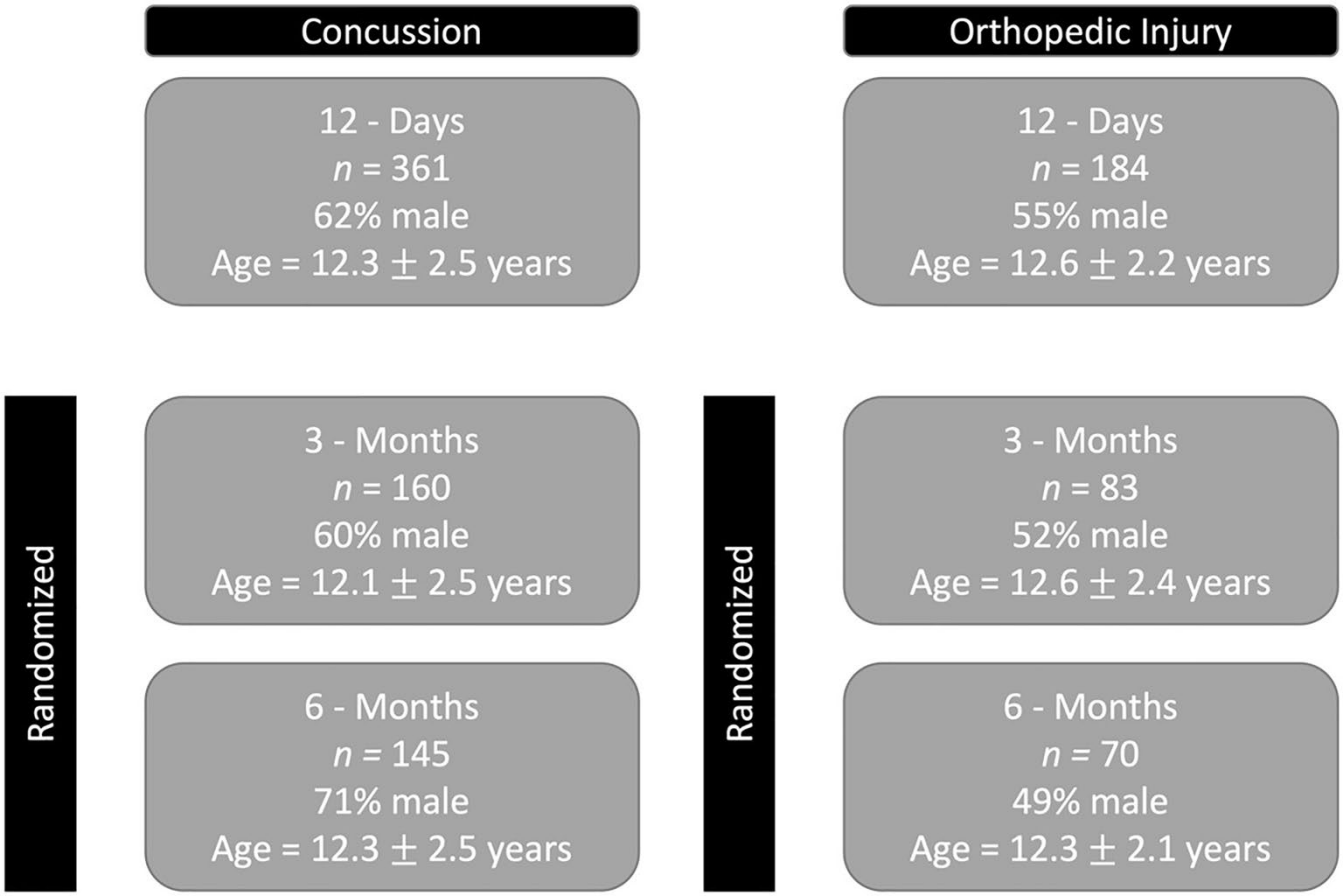
Demographics of concussion and orthopedic injury (OI) participants at each timepoint. Sex and age were not significantly different at the 12 day (*p* = 0.142, 0.201) and 3 month timepoints (*p* = 0.234, 0.225). Sex was significantly different between concussion and OI groups at 6 months = 0.0001. Age did not differ significantly at 6 months *p* = 0.925. Chi square was used to compare sex and t-tests for age.

Participant Demographics are shown in Fig. 2.

### Metabolite changes over 6 months following concussion

**Figure 3:**
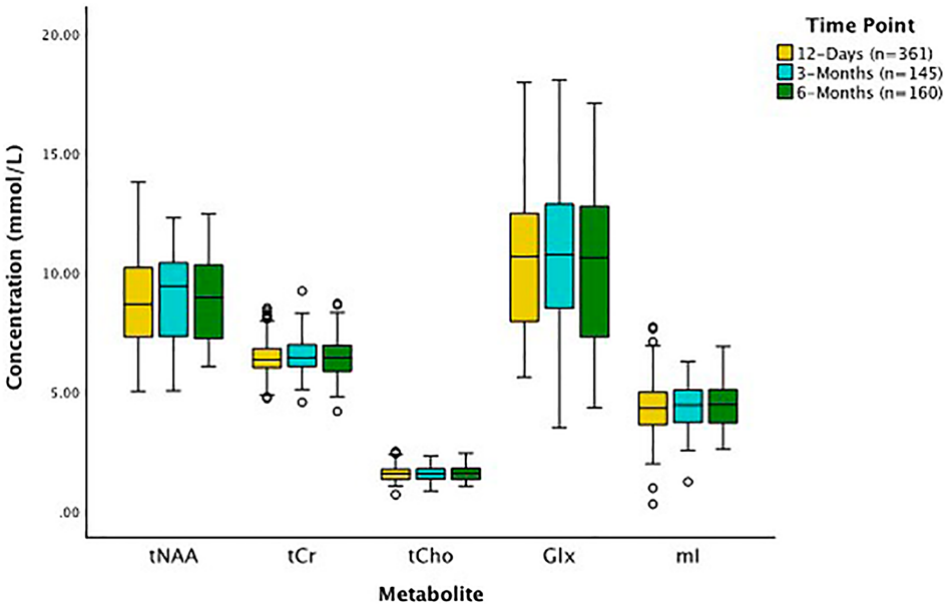
Concentration of tNAA, tCr, tCho, Glx, and mI at 12 days, 3 months, and 6 months post-concussion. All concussion participants had data collected at 12 days post-injury and were randomized to either the 3- or 6 month data collection. Linear mixed models for each metabolite controlling for age, sex, site, and vendor showed no significant effect of time for any metabolites. The black lines in the boxes represent the median and the box represents the interquartile range, with the 1.5 times the interquartile range values represented by the whiskers. The circles are statistical outliers (greater than 1.5 time the interquartile range from the median) but there were no data quality issues or reasons to exclude these spectra.

There were no significant changes in metabolite levels (tNAA, tCr, tCho, Glx, and mI) over time in pediatric concussion participants (*p* > 0.05, Fig. 3). Age was a significant covariate for tNAA and Glx; older children had higher tNAA (estimate 0.09 (0.02), *p* = 0.0001, t = 5.74) and lower Glx (estimate = − 0.13 (0.03), *p* = 0.0001, t = − 4.08).

**Figure 4:**
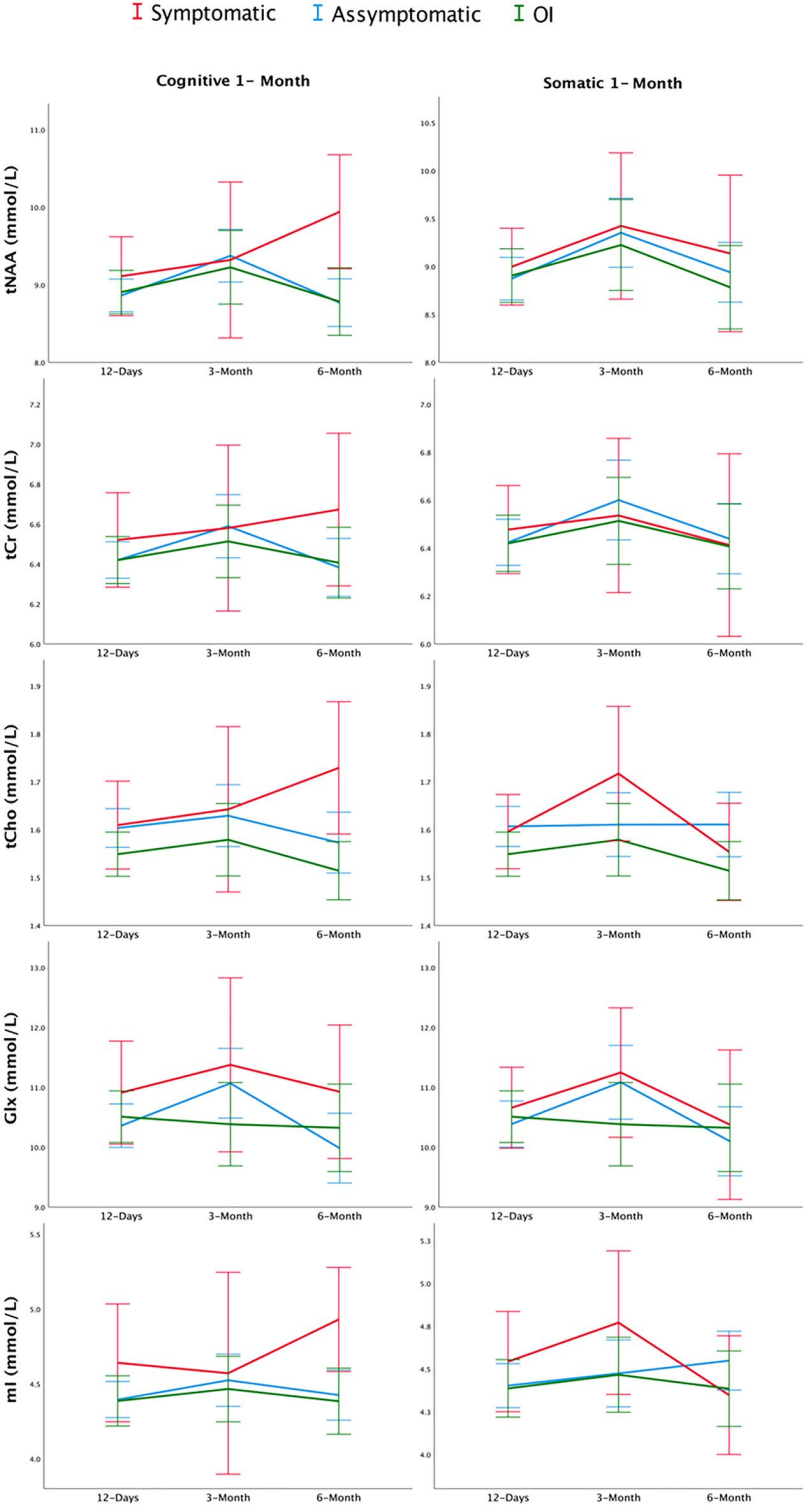
Line plots showing metabolite concentration (tNAA, tCr, tCho, Glx, and mI) for the three groups (symptomatic, asymptomatic, and OI) at the three timepoints studied (12 days, 3 months, and 6 months). Symptom status was determined by the HBI reliable change of cognitive or somatic summary scores at 4 weeks (1 month). Metabolite trajectories were not significantly different over time and were not significantly different between groups.

When comparing metabolite trajectories in the three groups (symptomatic and asymptomatic based on the 4 week timepoint and OI), no significant effects were found for group by timepoint interaction for any metabolite (*p* > 0.05). See Fig. 4 for metabolite trajectories by group with symptoms classified as cognitive or somatic based off the reliable change at 4 weeks.

### Cross-sectional metabolite differences at follow-up timepoints and the effect of symptom classification

**Figure 5:**
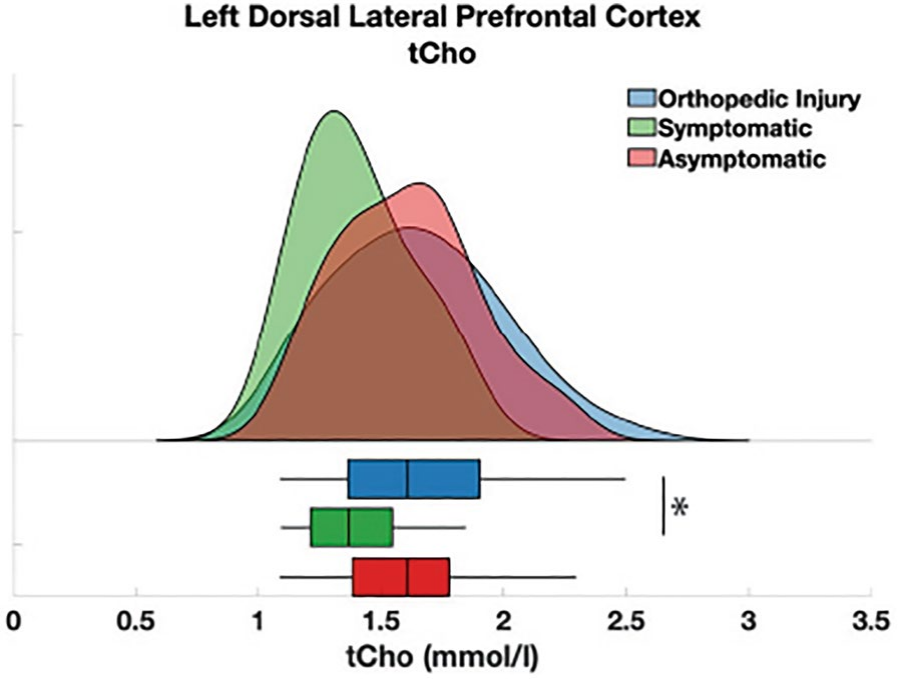
Mean tCho concentrations at 3 months separated into participants with concussion and somatic symptoms, those with concussion but without persisting somatic symptoms, and orthopedic injury (OI). Symptomatic and asymptomatic were defined at 3 months. The black lines in the boxes represent the median and the box represents the interquartile range, with the 1.5 times the interquartile range values represented by the whiskers. The asterisk indicates a significant group difference (*p* = 0.047) between OI and symptomatic groups.

Concussion participants classified as symptomatic according to somatic symptom scores at the 3-month timepoint had significantly lower tCho than children with OI at the 3-month assessment (mean difference (SE) = − 0.137(0.056), *p* = 0.047, η_p_^2^ = 0.029) (Fig. 5). This result would not survive multiple comparisons between different metabolites. No other group differences in metabolites were significant for either somatic or cognitive symptoms at 3- or 6 months (*p* > 0.05).

### Relationship between metabolites and acute or future symptoms in concussion participants

Linear regression models showed metabolite levels measured at 12 days in concussion participants (tNAA, tCr, tCho, Glx, and mI) were not associated with concussion symptoms (cognitive or somatic) at the 12 days timepoint (*p* > 0.05, Table 1).

**Table 1:** Results from the linear regression models examining the relationships between metabolites measured at 12 days and cognitive and somatic symptom burden at the 12 days assessment.

	HBI cognitive symptoms		HBI somatic symptoms	
	B (SE) =	*p* =	B (SE) =	*p* =
tNAA	− 0.826 (0.74)	0.267	− 0.342 (0.49)	0.481
tCr	0.599 (1.08)	0.581	0.193 (0.71)	0.785
tCho	− 1.74 (2.11)	0.41	0.568 (1.37)	0.68
Glx	− 0.09 (0.24)	0.709	− 0.004 (0.16)	0.981
mI	− 0.699 (0.78)	0.372	− 0.135 (0.51)	0.792
Age	0.154 (0.19)	0.418	0.587 (0.12)	**0.0001**
Sex	− 0.933 (0.91)	0.304	− 1.554 (0.59)	**0.009**
Site	0.09 (0.31)	0.774	0.334 (0.21)	0.104
Vendor	− 6.269 (2.21)	**0.005**	− 1.186 (1.44)	0.41

The model controls for age, sex, site, and vendor. Significant values are in bold.

**Figure 6:**
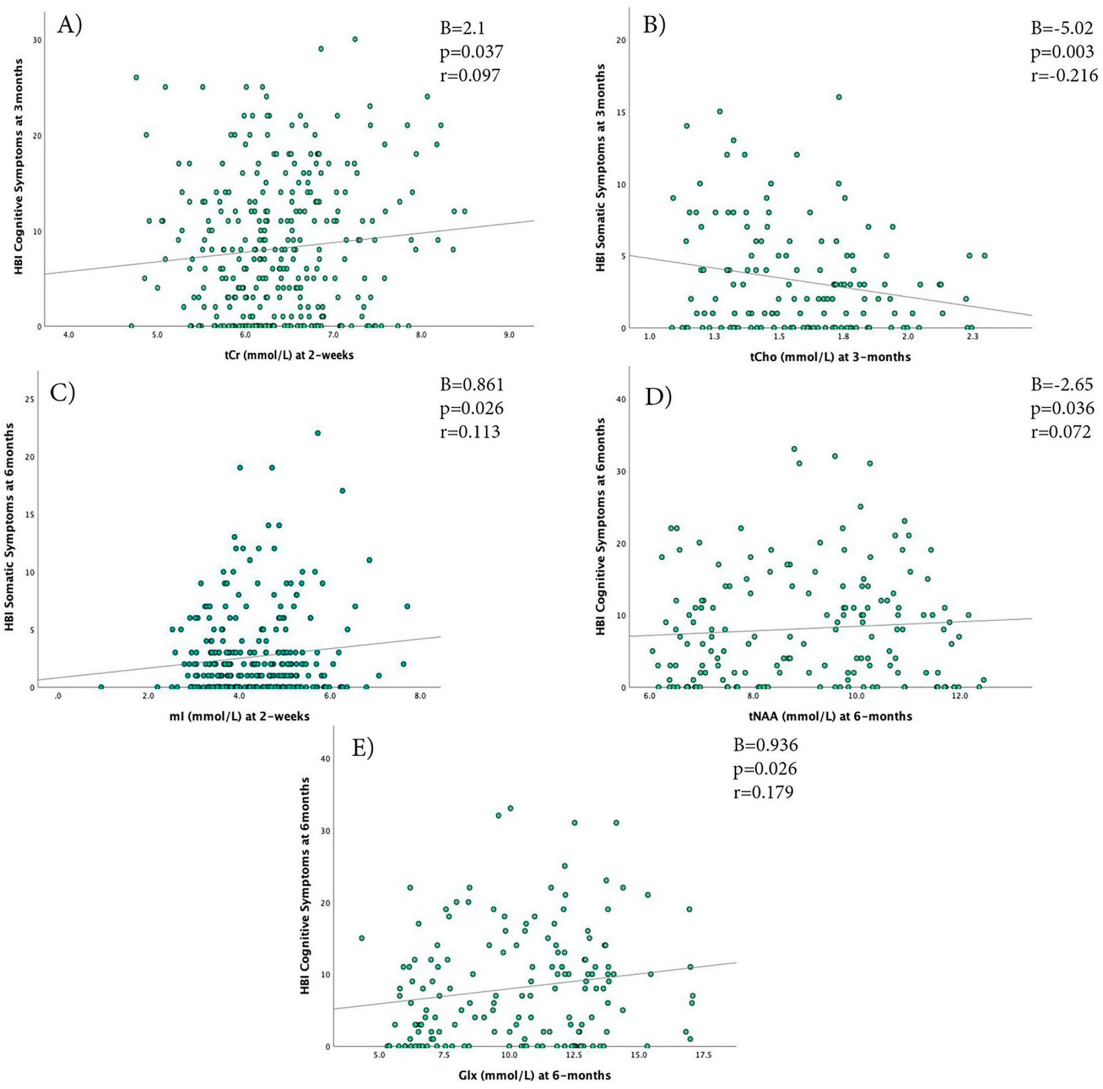
Scatterplot of the relationship of the metabolite concentrations that were found to have significant associations with HBI symptoms (Cognitive/Somatic) at 12 days, 3 months, and 6 months. (**A**) 12 day tCr levels and cognitive symptoms at 3 months, (**B**) 3 month tCho levels and somatic symptoms at 3 months, (**C**) 12 day mI levels and somatic symptoms at 6 months, (**D**) 6 month tNAA levels and 6 month cognitive symptoms, (**E**) 6 month Glx levels and 6 month cognitive symptoms.

Higher tCr at 12 days was significantly associated with higher cognitive symptoms at 3 months (B = 2.095, *p* = 0.037, r = 0.097, Fig. 6A). No other relations were significant between metabolites measured at 12 days and symptoms measured at 3 months (*p* > 0.05, Tables 2). Higher mI at 12 days was significantly positively associated with higher somatic symptoms at 6 months (B = 0.861, *p* = 0.026, r = 0.113, Table 3 and Fig. 6C). No other significant associations were found between metabolites measured at 12 days and symptoms measured at 6 months.

**Table 2:** Results from the linear regression models examining the relationship between metabolites measured at the 12 day assessment and cognitive and somatic symptom burden at the 3 month assessment.

	HBI cognitive symptoms		HBI somatic symptoms	
	B (SE) =	*p* =	B (SE) =	*p* =
tNAA	− 0.624 (0.68)	0.358	0.102 (0.38)	0.786
tCr	2.095 (1.0)	**0.037**	0.491 (0.55)	0.376
tCho	− 3.177 (2.0)	0.113	− 0.529 (1.12)	0.636
Glx	− 0.178 (0.23)	0.43	− 0.049 (0.13)	0.699
mI	− 0.652 (0.71)	0.362	0.042 (0.4)	0.915
Age	− 0.123 (0.18)	0.487	0.223 (0.1)	**0.024**
Sex	− 0.771 (0.85)	0.365	− 1.42 (0.47)	**0.003**
Site	− 0.133 (0.3)	0.653	0.105 (0.17)	0.523
Vendor	− 4.717 (2.02)	**0.02**	0.101 (1.14)	0.929

The model controls for age, sex, site, and vendor. Significant values are in bold.

**Table 3:** Results from the linear regression models examining the relationship of metabolites measured at the 12 days assessment and symptoms (cognitive and somatic) measured at the 6 month assessment.

	HBI cognitive symptoms		HBI somatic symptoms	
	B (SE) =	*p* =	B (SE) =	*p* =
tNAA	− 0.547 (0.75)	0.465	0.334 (0.37)	0.368
tCr	0.912 (1.09)	0.405	0.158 (0.55)	0.772
tCho	− 1.696 (2.14)	0.428	− 1.338 (1.06)	0.207
Glx	− 0.414 (0.24)	0.08	− 0.23 (0.12)	0.051
mI	− 0.227 (0.78)	0.771	0.861 (0.38)	**0.026**
Age	− 0.464 (0.19)	**0.013**	0.143 (0.09)	0.119
Sex	0.479 (0.9)	0.594	− 0.822 (0.44)	0.065
Site	− 0.074 (0.31)	0.815	0.081 (0.16)	0.602
Vendor	− 5.385 (2.17)	**0.014**	0.758 (1.08)	0.483

The model controls for age, sex, site, and vendor. Significant values are in bold.

Lower tCho at 3 months was significantly associated with higher somatic symptoms at 3 months (B = − 5.02, *p* = 0.003, r = − 0.216, Fig. 6B). This result survived multiple comparisons between different metabolites and between both symptom types. No other significant associations were found between metabolites measured at 3 months and symptoms measured at 3 months.

Lower tNAA at 6 months was significantly associated with higher cognitive symptoms at 6 months (B = − 2.65, *p* = 0.036, r = 0.072, Fig. 6D), and higher Glx at 6 months was significantly associated with higher cognitive symptoms at 6 months (B = 0.936, *p* = 0.026, 0.179, Fig. 6E). No other significant associations were found.

## Discussion

In the largest longitudinal study of MRS in pediatric concussion to date (545 participants in total, with over 200 participants at each timepoint in the study), we found that metabolites did not significantly change over time in the 6 months following concussion, nor were there differences between concussion or OI metabolite trajectories. Additionally, when the concussion group was divided into symptomatic and asymptomatic groups, we found that metabolite trajectories were not significantly different between groups or relative to OI controls. At the 3-month timepoint, however, the symptomatic and OI groups differed in tCho levels and 3-month tCho levels were associated with somatic symptoms at 3 months. Additional relationships were found between symptom burden and metabolite levels; but these were small in magnitude and may not be of clinical significance.

Two previous studies examined metabolites longitudinally across at least two timepoints in pediatric concussion. Maugans et al.^14^ performed measurements at 72 h, 14 days, and 30 days post-injury, and, consistent with our results, found no alterations to tNAA over time; they did not report on Cho and Cr results. The second longitudinal study, specific to sport-related pediatric concussion, found a linear increase in Glx/Cho and Ins/Cho in the thalamus over time (measurements at 72 h, 2 weeks, 1 year) in concussion participants^17^. Meyer and colleagues suggested that Glx and Ins only showed a significant increase as a ratio to Cho, not as individual concentrations^17^. This suggests that fluctuations in Cho concentration may have influenced these results, and the interpretation of Glx and Ins changes based off of the ratio.

As previously reported, no group differences were found at the 2 week timepoint in metabolites, even when exploring a variety of additional covariates (age, sex, time since injury, tissue composition)^11^. At 3 months post-concussion, we found that those with persisting somatic symptoms at 3 months had significantly lower tCho compared to OI. Consistent with this finding, we also found a significant relationship between tCho at 3 months and somatic symptom burden (Fig. 6B). As tCho can be interpreted as a marker of tissue cellularity, this suggests that at 3 months lower tCho could indicate lower cellular density^31^. Additionally, alterations in prefrontal gray matter volume have previously been related to stress-related somatic symptoms^32^. Therefore, cell damage as represented by tCho that results in altered tissue volume manifesting at 3 months post-injury may be associated with somatic symptoms. Our tCho result is consistent with a prior pediatric study that also found lower prefrontal tCho in sport-related concussion in comparison to controls at 3 months, despite no group differences at the acute (24–72 h) timepoint^16^.

At 3 months we found no other differences between those with and without persistent cognitive symptoms in comparison to OI controls, which contrasts results from Bartnik-Olson et al.^15^ who found a difference in NAA/Cr between pediatric concussion participants with cognitive complaints in comparison to healthy controls. An important methodological difference between the two studies is that Bartnik-Olson and colleagues had a healthy, non-injured comparison group whereas our study had an OI comparison group. An OI comparison group accounts for shared trauma experience, allowing conclusions about brain-injury specific effects. A study of children with concussion showed that the structural connectome of those with concussion differed to those of uninjured controls but not to those with OI, suggesting that some differences in brain imaging metrics reflect a general injury experience rather than brain-injury specific outcomes^33^.

Despite the difference in tCho at 3 months, we found no significant group differences in tNAA, tCr, Glx, and mI at the 3- and 6 month timepoint in comparison to OI. This too is consistent with previous studies in which NAA was comparable between concussion and control participants at 72 h, 12 days^17^, 3 months^16^, and 1 year^17^. Meyer and colleagues did find that Glx/Cho differed between concussion and controls at 12 day and 1 year timepoints. However, as described earlier, this result could be a product of opposing metabolite trends in Glx and Cho, where a change in Cho could have influenced the result rather than just a changing Glx signal. Another study examining concussion long after injury (> 1 year) found differences in tNAA in those with 1 previous history of concussion in comparison to those with 2 or more concussions and OI controls^18^.

In examining associations between metabolites at the 12 day timepoint and symptoms at later timepoints, tCr at 12 days was significantly positively associated with cognitive symptoms at 3 months and mI at 12 days was significantly associated with somatic symptoms at 6 months; however, these relationships were small in magnitude (r = 0.097 and 0.113, respectively). Higher tCr at 12 days may indicate an energy crisis that leads to later cognitive symptoms. Increased mI at 12 days may indicate glial dysfunction that leads to somatic symptoms long after injury. Decreased tNAA and increased Glx at 6 months were significantly associated with higher cognitive symptoms at 6 months, but the associations were small (r = 0.072, 0.179 respectively). Elevated tNAA and Glx may indicate increased bioenergetics and excitability that may have developed in response to cognitive dysfunction long after injury. This is similar to results from MacMaster et al.^18^ showing tNAA in the L-DLPFC was correlated with emotional distress > 1 year post-injury in those who had multiple previous concussions. There is little research examining the relationship between metabolites and symptom burden. This area of research warrants further exploration, and these results must be taken cautiously as the associations were weak and there were no group differences in metabolites at 12 days or 6 months.

### Strengths and limitations

The selection of OI as a comparison group is important as it is different from healthy control, or more specifically a non-injury comparison group. OI groups are used to differentiating brain injury specific effects from general injury response. A future direction would be to include a healthy control population to ensure differentiation between OI and healthy control groups in comparison to concussion. This study also takes advantage of multiple sites and has the largest longitudinal dataset in MRS in pediatric concussion and has sufficient power to detect metabolite change over time.

Concussion is a highly heterogenous condition, the division of the concussion participants into sub-groups based on symptom persistence helps to address this issue^10^. However, there is a relatively high degree of variability in metabolite levels at each timepoint. The high metabolite variability of the concussion group is unaffected by dividing the concussion group into symptomatic and asymptomatic groups as showed in Fig. 5. Additionally, the OI group may also have experienced variations in metabolites in response to the injury, whereas the majority of MRS in pediatric concussion literature has been conducted in comparison to a healthy population^14–17,34^, in which metabolite concentrations may be more homogeneous. As most concussion research is conducted in adults, the results are difficult to compare with the current literature. Children might have greater metabolite variability due to development and plasticity in the brain^10,11,35–39^, whereby greater variability in metabolites in pediatrics is necessary for an adaptable and compensatory response to injury. Concussion participants were randomized to either the 3- or 6 month follow-up sessions, this creates a discontinuity of each participant which may affect our ability to observe longitudinal metabolite changes or specific individual differences. It is, however, difficult to retain participants and increasing the number of measurements in large samples becomes cost prohibitive. The current study only looks at the L-DLPFC. As this study is limited to this area, and due to the heterogeneity of concussion, we cannot make conclusions regarding whole brain effects. Future studies could use multi-voxel magnetic resonance spectroscopic imaging (MRSI) to explore different brain regions as well as whole brain effects of injury.

## Conclusion

In the largest longitudinal cohort of MRS in pediatric concussion to date, we found that metabolites in a concussion population do not change over time, however, there appears to be tCho changes at 3 months that are related to somatic symptoms. Future work should further explore concussion sub-groups not identified in the current study to better characterize different injury trajectories and the impact of symptoms.

### Supplementary Information

Supplementary Information.

## Data Availability

A dataset with deidentified participant data and a data dictionary will be made available upon reasonable request from any qualified investigator, subject to a signed data access agreement. Please contact the corresponding author PL for details.
