# Supplementary material for: Longitudinal changes in brain metabolites following pediatric concussion
Source: Sci Rep. 2024 Feb 8;14:3242. doi: 10.1038/s41598-024-52744-7 (PMC10853495; doi:10.1038/s41598-024-52744-7)
Supplement: Supplementary file 1 — Supplementary Information. [file 41598_2024_52744_MOESM1_ESM.docx]

**Longitudinal changes of brain metabolites following pediatric concussion: An Advancing Concussion Assessment in Pediatrics (A-CAP) study**

**Authors:** **Parker L La^1, 2, 3*^**, **Robyn Walker^1, 2, 3^,** **Tiffany K Bell^1, 2, 3^**, **William Craig^4^, Quynh Doan^5^, Miriam H. Beauchamp^6^, Roger Zemek^7, 8^, Keith Owen Yeates^9^, Ashley D Harris^1, 2, 3^** on behalf of the Pediatric Emergency Research Canada A-CAP study team

^1^*Department of Radiology, University of Calgary, Calgary, Alberta, Canada; Alberta*

^2^*Children’s Hospital Research Institute, Calgary, Alberta, Canada*

^3^ *Hotchkiss Brain Institute, University of Calgary, Calgary, Alberta, Canada*

^4^*Department of Pediatrics, University of Alberta and Stollery Children’s Hospital, Edmonton, Alberta, Canada*

^5^*Department of Pediatrics, University of British Columbia and BC Children’s Hospital Research Institute, Vancouver, BC, Canada*

^6^*Department of Psychology, University of Montreal and Ste Justine Hospital Research Centre, Montreal, Quebec, Canada*

^7^*Department of Pediatrics and Emergency Medicine, Children’s Hospital of Eastern Ontario, University of Ottawa, Ottawa, Ontario, Canada*

^8^*Childrens’ Hospital of Eastern Ontario Research Institute; University of Ottawa, Ottawa, Ontario, Canada*

^9^*Department of Psychology, University of Calgary, Calgary, Alberta, Canad*

*Corresponding Author - Parker L La email: parker.la@ucalgary.ca

Supplementary Materials:

**Supplementary Table 1.** Minimum reporting standards for in vivo magnetic resonance spectroscopy studies.

| Site (Name or Number) | Calgary | Edmonton | Montreal 1 | Montreal 2 | Ottawa | Vancouver |
| --- | --- | --- | --- | --- | --- | --- |
| 1. Hardware |  |  |  |  |  |  |
| a. Field strength [T] | 3 | 3 | 3 | 3 | 3 | 3 |
| b. Manufacturer | GE | Siemens | GE | Siemens | Siemens | GE |
| c. Model (software version if available) | GE MR750w | Siemens Prisma | GE MR750 | Siemens Prisma | Siemens Skyra | GE MR750 |
| d. RF coils: nuclei (transmit/ receive), number of channels, type, body part | ^1^H, 32-channel head coil | ^1^H, 64-channel head/neck coil | ^1^H, 32-channel head coil | ^1^H, 64-channel head coil | ^1^H, 64-channel head coil | ^1^H, 32-channel head coil |
| e. Additional hardware | No | No | No | No | No | No |
| 2. Acquisition |  |  |  |  |  |  |
| a. Pulse sequence | Vendor-supplied PRESS | Vendor-supplied PRESS | Vendor-supplied PRESS | Vendor-supplied PRESS | Vendor-supplied PRESS | Vendor-supplied PRESS |
| b. Volume of Interest (VOI) locations | Left Dorsallateral prefrontal cortex | Left Dorsallateral prefrontal cortex | Left Dorsallateral prefrontal cortex | Left Dorsallateral prefrontal cortex | Left Dorsallateral prefrontal cortex | Left Dorsallateral prefrontal cortex |
| c. Nominal VOI size [cm^3^] | 222  | 222 | 222 | 222 | 222 | 222 |
| d. Repetition Time (TR), Echo Time (TE) [ms] | 2000/30 | 2000/30 | 2000/30 | 2000/30 | 2000/30 | 2000/30 |
| e. Total number of Excitations or acquisitions per spectrum  In time series for kinetic studies   1. Number of Averaged spectra (NA) per time-point 2. Averaging method (e.g. block-wise or moving average) 3. Total number of spectra (acquired / in time-series) | 96 averages with a 8 step phase cycle | 96 averages with a 8 step phase cycle | 96 averages with a 8 step phase cycle | 96 averages with a 8 step phase cycle | 96 averages with a 8 step phase cycle | 96 averages with a 8 step phase cycle |
| f. Additional sequence parameters  (spectral width in Hz, number of spectral points, frequency offsets) | 5000 Hz, n=4096 | 2000 Hz, n=2048 | 5000 Hz, n=4096 | 2000 Hz, n=2048 | 2000 Hz, n=2048 | 5000 Hz, n=4096 |
| g. Water Suppression Method | CHESS (vendor provided) | WET (vendor provided) | CHESS (vendor provided) | WET (vendor provided) | WET (vendor provided) | CHESS (vendor provided) |
| h. Shimming Method, reference peak, and thresholds for “acceptance of shim” chosen | Automated B0 field mapping (vendor provided). No threshold was specified. | Automated B0 field mapping (vendor provided). No threshold was specified. | Automated B0 field mapping (vendor provided). No threshold was specified. | automated B0 field mapping (vendor provided). No threshold was specified. | Automated B0 field mapping (vendor provided). No threshold was specified. | Automated B0 field mapping (vendor provided). No threshold was specified. |
| i. Triggering or motion correction method  (respiratory, peripheral, cardiac triggering, incl. device used and delays) | N/A | N/A | N/A | N/A | N/A | N/A |
| 3. Data analysis methods and outputs |  |  |  |  |  |  |
| a. Analysis software | LCModel 6.3-1J | LCModel 6.3-1J | LCModel 6.3-1J | LCModel 6.3-1J | LCModel 6.3-1J | LCModel 6.3-1J |
| b. Processing steps deviating from quoted reference or product | Data pre-processed through FID-A | Data converted to .RAW format from FID-A prior to quantification in LCModel | Data pre-processed through FID-A | Data converted to .RAW format from FID-A prior to quantification in LCModel | Data converted to .RAW format from FID-A prior to quantification in LCModel | Data pre-processed through FID-A |
| c. Output measure  (e.g. absolute concentration, institutional units, ratio)Processing steps deviating from quoted reference or product | Absolute Concentration | Absolute Concentration | Absolute Concentration | Absolute Concentration | Absolute Concentration | Absolute Concentration |
| d. Quantification references and assumptions, fitting model assumptions | Custom basissets based on vendor-specific waveforms, generated from FID-A | Custom basissets based on vendor-specific waveforms, generated from FID-A | Custom basissets based on vendor-specific waveforms, generated from FID-A | Custom basissets based on vendor-specific waveforms, generated from FID-A | Custom basissets based on vendor-specific waveforms, generated from FID-A | Custom basissets based on vendor-specific waveforms, generated from FID-A |
| 4. Data Quality |  |  |  |  |  |  |
| a. Reported variables  (SNR, Linewidth (with reference peaks)) | See data description in: La et al. Comparison of different approaches to manage multi-site magnetic resonance spectroscopy clinical data analysis. Front Psychol. 2023 Apr 20;14:1130188. doi: 10.3389/fpsyg.2023.1130188. | | | | | |
| b. Data exclusion criteria | Excessive motion, low SNR, high linewidth | Excessive motion, low SNR, high linewidth | Excessive motion, low SNR, high linewidth | Excessive motion, low SNR, high linewidth | Excessive motion, low SNR, high linewidth | Excessive motion, low SNR, high linewidth |
| c. Quality measures of postprocessing Model fitting (e.g. CRLB, goodness of fit, SD of residual) | CRLB, SNR, Linewidth | CRLB, SNR, Linewidth | CRLB, SNR, Linewidth | CRLB, SNR, Linewidth | CRLB, SNR, Linewidth | CRLB, SNR, Linewidth |
| d. Sample Spectrum | Yes, figure 1 | No, see supplementary figure in: La et al. Comparison of different approaches to manage multi-site magnetic resonance spectroscopy clinical data analysis. Front Psychol. 2023 Apr 20;14:1130188. doi: 10.3389/fpsyg.2023.1130188.  .  . | | | | |
